# Ultrasound-Assisted Extraction, Identification, and Quantification of Antioxidants from ‘Jinfeng’ Kiwifruit

**DOI:** 10.3390/foods11060827

**Published:** 2022-03-14

**Authors:** Ying-Hui Mai, Qi-Guo Zhuang, Qiao-Hong Li, Kui Du, Ding-Tao Wu, Hua-Bin Li, Yu Xia, Fan Zhu, Ren-You Gan

**Affiliations:** 1 China-New Zealand Belt and Road Joint Laboratory on Kiwifruit, Sichuan Provincial Academy of Natural Resource Sciences, Chengdu 610213, China; myin315@aucklanduni.ac.nz (Y.-H.M.); xqrenjia@126.com (Q.-H.L.); dk170502@163.com (K.D.); 2 Research Center for Plants and Human Health, Institute of Urban Agriculture, Chinese Academy of Agricultural Sciences, Chengdu 610213, China; xiayu01@caas.cn; 3 School of Chemical Sciences, University of Auckland, Auckland 1142, New Zealand; f.zhu@auckland.ac.nz; 4 Kiwifruit Breeding and Utilization Key Laboratory of Sichuan Province, Sichuan Provincial Academy of Natural Resource Sciences, Chengdu 610213, China; 5 Key Laboratory of Coarse Cereal Processing (Ministry of Agriculture and Rural Affairs), Sichuan Engineering & Technology Research Center of Coarse Cereal Industrialization, School of Food and Biological Engineering, Chengdu University, Chengdu 610106, China; wudingtao@cdu.edu.cn; 6 Guangdong Provincial Key Laboratory of Food, Nutrition and Health, Department of Nutrition, School of Public Health, Sun Yat-sen University, Guangzhou 510080, China; lihuabin@mail.sysu.edu.cn

**Keywords:** *Actinidia chinensis*, green extraction, antioxidant, polyphenols, vitamin C

## Abstract

Kiwifruit (*Actinidia chinensis*) is a nutrient-dense fruit abundant in vitamin C and phenolic compounds, and it exhibits strong antioxidant capacity. However, the antioxidants in ‘Jinfeng’ kiwifruit have seldom been extracted and analyzed, and the conditions for the extraction of kiwifruit antioxidants by ultrasound-assisted extraction (UAE) have seldom been investigated. In this study, response surface methodology (RSM) was used to optimize UAE conditions to extract antioxidants from ‘Jinfeng’ kiwifruit. In addition, the antioxidant capacity, contents of total phenolics and total flavonoids, ascorbic acid, and the profiles of antioxidants were also analyzed. The results showed that the optimal UAE conditions included 68% ethanol, liquid/solid ratio at 20 mL/g, extraction time at 30 min, extraction temperature at 42 °C, and ultrasonic power at 420 W. Under these conditions, the ABTS value of kiwifruit was 70.38 ± 1.38 μM TE/g DW, which was 18.5% higher than that of the extract obtained by conventional solvent extraction. The total phenolic and flavonoid contents were 15.50 ± 0.08 mg GAE/g DW and 5.10 ± 0.09 mg CE/g DW, respectively. Moreover, 20 compounds were tentatively identified by UPLC–MS/MS, and the content of main compounds, such as procyanidin B2, neochlorogenic acid, and epicatechin, were determined by HPLC–DAD. This research revealed the profiles of antioxidant phytochemicals in ‘Jinfeng’ kiwifruit, which can be a good dietary source of natural antioxidants with potential health functions.

## 1. Introduction

Kiwifruit (*Actinidia*) is an edible berry reputed for its remarkable nutritional value and attractive taste [1]. Up to now, more than 70 species and 120 taxa have been recognized globally, including *A. deliciosa*, *A. chinensis*, *A. arguta*, *A. kolomikta*, *A. eriantha*, *A. macrosperma*, and *A. polygama* [2]. However, only a few cultivars from *A. chinensis*, *A. deliciosa*, *A. arguta*, and *A. eriantha* have been commercially cultivated by the international kiwifruit industry [3]. Plenty of research has documented that consuming kiwifruit has a large number of health benefits, such as antioxidant [1,4], anti-inflammatory [5,6], anti-cancer [7], anti-obesity [8] and gastrointestinal function-improving [9,10] capacities. These health benefits can be ascribed to the abundant bioactive compounds in kiwifruits, such as vitamins, polysaccharides, organic acids, and polyphenols. Vitamin C is the most significant nutrient attribute of kiwifruit [11]. A previous review has compared the total ascorbic acid content of two commercial kiwifruit cultivars with some common fruits, and the ascorbic acid content of kiwifruit is two to three times higher than that of strawberry, orange, and pineapple [11]. Moreover, kiwifruit exceeds the recommended daily intake by the European Union (80 mg/100 g), which means that consuming 90 g of green kiwifruit or 50 g of SunGold kiwifruit per day can fulfil an adult’s daily vitamin C requirement [11]. Organic acids are important in fruits, affecting their maturity development, flavour and taste, and utilization [12,13]. Common organic acids like malic acid, citric acid, and quinic acid can be found in kiwifruit, even if their contents vary among species and cultivars [14]. In addition, phenolic compounds consisting of phenolic acids, flavonoids, and coumarins, have gained more attention in recent years because of their related bioactivities [15]. According to previous literature, caffeic acid, chlorogenic acid, catechin, procyanidin B2, rutin, and quercetin have been widely identified and regarded as major phenolic compounds in kiwifruit [15,16]. However, the types and the contents of the phenolic compounds are varied by species and cultivar, maturity stage, processing technique, and extraction method [16].

Conventional solvent extraction (CSE) is the most common method for extracting antioxidants from plants [17]. However, it has several drawbacks, such as consuming a long time, wasting a large number of solvents and energy, using toxic solvents, and degrading thermally unstable compounds [17,18]. Therefore, several green and novel techniques, such as ultrasound-assisted extraction (UAE), microwave-assisted extraction (MAE), enzyme-assisted extraction (EAE), deep eutectic solvent extraction, and supercritical fluid extraction, have been developed for extracting bioactive compounds from plants [19,20,21,22]. Among these techniques, UAE shows the advantages in time and energy savings, toxic organic solvent reduction, and easy operation [23,24]. Ultrasonic acoustic cavitation can tear plant cells and accelerate the release and dispersion of intracellular bioactive compounds into the solvents, which increases the extraction efficiency [23].

To our knowledge, the antioxidants in ‘Jinfeng’ kiwifruit have never been extracted and analyzed before. In addition, although some studies reported extracting the phenolic antioxidants from kiwifruit using UAE [25,26], the effects of the extraction conditions have seldom been optimized. Therefore, this study was conducted to establish an optimized method to extract antioxidants in ‘Jinfeng’ kiwifruit. Meanwhile, the antioxidant compounds extracted under the optimized conditions were further tentatively identified by UPLC–MS/MS, and the main compounds were quantified by HPLC–DAD.

## 2. Materials and Methods

### 2.1. Chemicals and Reagents

Formic acid and 2,2′-azinobis(3-ethyl-benzothiazoline-6-sulfonic acid) diammonium salt (ABTS) was purchased from Aladdin Biochemical Technology Co., Ltd. (Shanghai, China). 6-hydroxy-2,5,7,8-tetramethylchromane-2-carboxylic acid (Trolox) was provided by Sigma-Aldrich Chemical Co., Ltd. (St. Louis, MO, USA). Potassium persulfate was procured from Macklin Biochemical Co., Ltd. (Shanghai, China). Ethanol and acetonitrile were obtained from Kelong Chemical Factory (Chengdu, China). Gallic acid, catechin, ascorbic acid, neochlorogenic acid, chlorogenic acid, caffeic acid, epicatechin, procyanidin B1, procyanidin B2, quercetin 3-*O*-glucoside, and quercetin 3-*O*-rhamnoside were provided by Madsen Technology Co., Ltd. (Chengdu, China). All chemicals were of analytical grade. Deionized water was applied to all experiments.

### 2.2. Sample Preparation

Yellow-fleshed ‘Jinfeng’ kiwifruit (*Actinidia chinensis*) was collected from Shifang Kiwifruit Research Station, Sichuan Provincial Academy of Natural Resource Sciences, Sichuan, China, when they reached commercial maturity. The kiwifruit was washed by water thoroughly and peeled by hand using a household fruit peeler. The peeled kiwifruit was sliced into 2–3 mm-thick slices and immediately freeze-dried for 72 h. The lyophilized slices were then ground into fine powders by a laboratory pulverizer and passed through a 60-mm sieve. The powders were sealed in centrifuged tubes and stored at −20 °C for further experiments.

### 2.3. Experimental Design

A single-factor experiment was carried out to investigate the effects of different extraction parameters on extraction efficiency and to determine a narrow range of different parameters. Five extraction parameters were studied, which included the concentration of ethanol (50, 60, 70, 80, and 90%), liquid/solid (L/S) ratio (10, 15, 20, 25, and 30 mL/g), extraction time (20, 30, 40, 50, and 60 min), extraction temperature (20, 30, 40, 50, and 60 °C), and ultrasonic power (360, 420, 480, 560, and 600 W). According to the results of the single-factor experiment, three main variables were selected for the following RSM optimization.

Design Expert Software (version 8.0.6) was used to generate a Box–Behnken design and analyze the data. A 3-level, 3-factor Box–Behnken design was generated by the software, consisting of 17 experimental runs. Each variable was coded at 3 levels (−1, 0, 1). The experimental data were analyzed by fitting into the following second-order polynomial model:

(1) $$Y=\beta_{0}+\sum \beta_{i}X_{j}+\sum \beta_{ii}X_{i}{}^{2}+\sum \beta_{ij}X_{i}X_{j}$$

In Equation (1), *Y* stands for the response value (the observed value of ABTS assay), *X_i_* and *X_j_* represent diverse independent variables, *β*_0_ is the intercept, and *β_i_*, *β_j_*, and *β_ij_* represent the linear, quadratic, and interaction regression coefficients, respectively.

### 2.4. Extraction Assays

#### 2.4.1. Ultrasound-Assisted Extraction (UAE)

UAE was applied according to the previous study with modifications [24]. Briefly, a kiwifruit sample (1.0 g) was placed in a 50 mL tube and mixed with various volumes of aqueous ethanol according to the experimental design. Then, the sample was extracted in an ultrasonic bath, with other parameters (extraction time, extraction temperature, and ultrasound power) controlled based on the experimental design. After that, the mixture was centrifuged (3000× *g*, 20 °C, 20 min), and the supernatant was collected and stored at 4 °C.

#### 2.4.2. Conventional Solvent Extraction (CSE)

In order to compare the extraction efficiency of UAE, a CSE was conducted under the obtained optimal UAE extraction conditions without ultrasound. Briefly, the kiwifruit sample (1.0 g) was extracted by 10 mL of 68% ethanol solution using a shaking air bath at 42 °C for 30 min. After extraction, the supernatant was collected after centrifugation (3000× *g*, 20 °C, 20 min) and then stored at 4 °C.

### 2.5. Determination of Antioxidant Capacity

An ABTS assay was employed to evaluate the antioxidant capacity of the kiwifruit extracts with slight modifications from a previous method [27]. In brief, 7 mM of ABTS stock solution was mixed with 2.45 mM of potassium persulphate (K_2_S_2_O_8_) in a volume ratio of 1:1. The mixed solution was incubated in the dark at room temperature for 16 h and used within 24 h. The absorbance of the incubated mixture was adjusted to 0.700 ± 0.05 with water before use. The adjusted ABTS working solution (3.9 mL) was added to 100 μL of the adequately diluted kiwifruit extract, mixed well, and kept in the dark at room temperature for 6 min. Finally, the absorbance was measured at 734 nm against the blank, which was replaced by the extraction solvent, using an ultraviolet-visible (UV-Vis) spectrophotometer. Trolox was used as the standard with concentrations of 50–800 μM. The data were expressed as μM Trolox/g dry weight (DW) of kiwifruit samples.

### 2.6. Determination of Total Phenolic Content and Total Flavonoid Content

Total phenolic content (TPC) was determined by Folin–Ciocalteu colourimetry following the method described by [28]. Briefly, the Folin–Ciocalteu working solution was diluted 10-fold with the Folin–Ciocalteu stock reagent using water. A quantity of 2 mL of the Folin–Ciocalteu working solution was added to 400 μL of the properly diluted kiwifruit extract, and the mixture was kept at room temperature for 4 min. Then, sodium carbonate solution (75 g/L, 1.6 mL) was added to the mixture, and it was incubated in the dark at room temperature for 2 h. The absorbance was measured at 760 nm, and gallic acid (0–0.1 mg/mL) was used as the standard. The results were recorded as mg of gallic acid equivalents per g dry weight (mg GAE/g DW) of kiwifruit.

Total flavonoid content (TFC) was determined by AlCl_3_-based colourimetry adapted from a previous study [29]. In short, 3.5 mL of Milli-Q water was mixed well with 0.5 mL of the properly diluted kiwifruit extract. After that, 150 μL of 5% sodium nitrite (NaNO_2_) solution was added to the mixture to react for 6 min, and 150 μL of 10% aluminium chloride (AlCl_3_) solution was added to the mixture to react for another 6 min. Finally, 1 mL of 0.1 M sodium hydroxide (NaOH) solution was added and mixed well. The absorbance was recorded at 510 nm. Catechin solutions at different concentrations (0.05–0.4 mg/mL) were used to calculate the standard curve. The data were expressed as mg catechin equivalents per g dry weight (mg CE/g DW) of kiwifruit.

### 2.7. Identification of Antioxidant Phytochemicals in Kiwifruit by UPLC–MS/MS

Ultra-high performance liquid chromatography-tandem mass spectrometry (UPLC–MS/MS) analysis was performed on a Thermo Scientific SII System (San Jose, CA, USA). A Hypersil GOLD column (2.1 × 100 mm, 1.9 μm) was used for chromatographic separation. The mobile phase consisted of 0.5% formic acid–water solution (solvent A) and acetonitrile (solvent B) in this experiment. The column was eluted with a gradient of 5% B (0–5 min), 5–7% B (5–10 min), 7–10% B (10–20 min), 10–30% B (20–40 min), 30–95% B (40–45 min), 95% B (45–50 min), 95–5% B (50–51 min), and 5% B (51–56 min). The column temperature, flow rate, and injection volume were 30 °C, 0.3 mL/min, and 5 μL, respectively. The electrospray ionization (ESI) of the Q-Exactive Focus mass spectrometer, which has an HCD (higher-energy collisional dissociation) cell, was operated in the negative mode. The scanning range of the orbitrap mass analyzer was set at m/z 100–1000, with the resolution at 70,000 in negative polarity. The linear ion-trap analyzer used an ion isolation window of ±1.0 m/z and 20, 40, and 60 NCE (normalized collision energy) to analyze the MS. System operation and data acquisition were conducted using Xcalibur 4.0 software (Thermo Scientific, San Jose, CA, USA). The analysis of phytochemicals was carried out by comparing the parent and fragment ions with the MassBank database (https://massbank.eu/MassBank/ (accessed on 12 July 2021)), and subsequently by comparing with the previous literature.

### 2.8. Quantification of Main Phytochemicals in Kiwifruit by HPLC

The contents of ascorbic acid and major phenolic compounds in the kiwifruit were determined using an Agilent 1260Ⅱ HPLC system (Palo Alto, CA, USA), coupled with a diode-array detector (DAD). The Agilent Zorbax SB-C18 column (4.6 mm × 150 mm, 5 μm) was used to separate the samples. For the ascorbic acid, the method was adapted from [29]. Briefly, the mobile phase consisted of 0.1% formic acid (solvent A) and acetonitrile (solvent B), and the column temperature, injection volume, and flow rate were set at 30 °C, 10 µL, and 0.8 mL/min, respectively. The separation program was maintained at 1% B for the first 5 min, then reached 90% B in 1 min, kept constant for the next 6 min, and finally returned to 1% B within the following 5 min. A wavelength of 245 nm was selected to determine the ascorbic acid content, and the L-ascorbic acid (0.01–0.1 mg/mL) was determined as the standard. The results were expressed as mg of L-ascorbic acid equivalents per g dry weight (mg L-AA/g DW) of kiwifruit. For the major phenolic compounds, the mobile phases consisted of a 0.5% formic acid–water solution (solvent A) and acetonitrile (solvent B). The program of the separation with the flow rate of 0.8 mL/min was based on [30] with some modifications, and the program was as follows: 0–5 min, 5% B; 5–10 min, 5–7% B; 10–20 min, 7–10% B; 20–40 min, 10–30% B; 40–45 min, 30–95% B; 45–50 min, 95% B; 50–51 min, 95–5% B; 51–56 min, 5% B. The injection volume and the column temperature were set at 10 μL and 30 °C, respectively. The hydroxybenzoic acids and flavan-3-ols were detected at 280 nm; the hydroxycinnamic acids were detected at 320 nm; and the flavonols were detected at 360 nm. Nine standards, including three hydroxycinnamic acids (neochlorogenic acid, chlorogenic acid, and caffeic acid), four flavan-3-ols (catechin, epicatechin, procyanidin B1, and procyanidin B2), and two flavonols (quercetin 3-*O*-glucoside and quercetin 3-*O*-rhamnoside), were used for the quantification of the phenolic compounds in the kiwifruit. The content of individual phenolic compounds was expressed as mg per g dry weight (μg/g DW) of kiwifruit.

### 2.9. Statistical Analysis

All measurements were performed in triplicate, and the data were expressed as mean ± standard deviations (SD). The results were subjected to one-way analysis of variance (ANOVA) using SPSS 18.0 software (IBM, Chicago, IL, USA). Means were compared using Duncan’s multiple range test, and the statistical significance was defined at *p*-values < 0.05.

## 3. Results and Discussion

### 3.1. Results of Single-Factor Experiment

During the ultrasonic-assisted extraction, the extraction efficiency could be affected by multiple parameters, such as the concentration of the extraction solvent, the ratio of liquid/solid, extraction time, extraction temperature, and the power of ultrasound. Hence, a single-factor experiment was conducted to estimate three critical parameters for further optimization.

#### 3.1.1. The Effects of Ethanol Concentration

**Figure 1:**
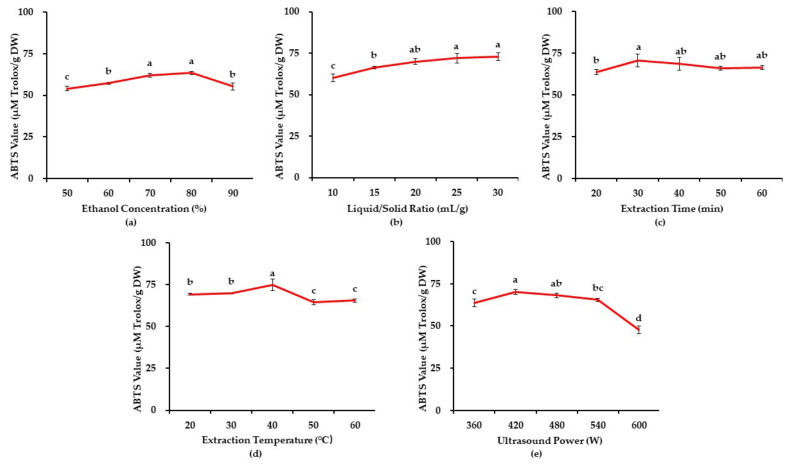
Effects of ethanol concentration (**a**); liquid/solid ratio (**b**); extraction time (**c**); extraction temperature (**d**); and ultrasound power (**e**) on ABTS-reducing capacities of ‘Jinfeng’ kiwifruit. Varying lowercase letters (a–d) indicate statistically significant differences (*p* < 0.05).

Ethanol, methanol, and cold acetone are usually used as solvents to extract bioactive compounds from different parts of kiwifruit [7,31,32]. Since ethanol is the most eco-friendly solvent among the solvents mentioned above, aqueous ethanol was selected for this study [33]. The effects of different ethanol concentrations (50, 60, 70, 80, and 90%) on the extraction efficiency were determined. Meanwhile, other extraction parameters were kept constant, including an L/S ratio of 20 mL/g, extraction temperature of 40 °C, extraction time of 30 min, and ultrasonic power of 480 W. The ABTS assay evaluated the antioxidant activity of the extracts. Figure 1a shows that the ABTS value increased from 20% to 80% and then decreased slightly at 90%. Though the highest ABTS value was observed at 80%, there was no statistically significant difference between that at 70% and 80%. In consideration of consuming fewer organic solvents and reducing the harm to the environment, a 70% ethanol solution was selected for the following studies.

#### 3.1.2. The Effects of Liquid/Solid Ratio

The L/S ratio ranging from 10 to 30 mL/g was investigated. At the same time, other extraction parameters were controlled as follows: ethanol concentration of 70%, extraction temperature of 40 °C, extraction time of 40 min, and ultrasound power of 480 W. As shown in Figure 1b, the antioxidant capacity increased rapidly from 10 to 20 mL/g, while there was no significant change as the L/S ratio increased higher than 20 mL/g. The increasing trend from 10 to 20 mL/g might be due to the concentration difference becoming more remarkable as the L/S ratio increased, which accelerated the mass transfer and the diffusion of antioxidants into the medium. However, the yield of antioxidants could hardly be enhanced after the maximum mass transfer was reached [34]. Similar results were consistent with previous studies [27,35]. In order to lower the extraction cost, 20 mL/g was finally chosen as the optimized L/S ratio since there was no statistical significance from 20 to 30 mL/g.

#### 3.1.3. The Effects of Extraction Time

The effects of extraction time ranging from 20 to 60 min on extraction efficiency were investigated, with other extraction parameters controlled as follows: ethanol concentration of 70%, L/S ratio of 20 mL/g, extraction temperature of 40 °C, and ultrasound power of 480 W. The results showed that the ABTS value increased with a rise in temperature and reached the highest point at 30 °C. However, as the temperature continued to rise, the ABTS value showed a slightly decreasing tendency (Figure 1c). The possible explanation for the above results is that the ultrasound treatment facilitated the degradation of cell walls, which accelerated the release of the antioxidants inside the cells [23]. However, long-term extraction would have also led to the degradation of the antioxidants, which caused the decrease in the ABTS value [36]. Thus, 30 min was the optimal extraction time.

#### 3.1.4. The Effects of Extraction Temperature

The effect of extraction temperature was determined in the range of 20–60 °C, with other parameters constant as follows: the concentration of ethanol of 70%, the ratio of L/S of 20 mL/g, the extraction time of 30 min, and the ultrasonic power of 480 W. Figure 1d shows that the antioxidant capacity rose mildly from 20 °C to 40 °C, and then witnessed a slight reduction. A possible reason is that the acoustic cavitation induced by ultrasonic treatment and the molecular movement would be enhanced by increasing the temperature. However, the ultrasonic wave could induce a high temperature, causing a surface tension reduction, raising the vapour pressure in microbubbles, and degrading some temperature-sensitive antioxidants, which would reduce the yield of antioxidants [37,38]. Therefore, 40 °C was selected for subsequent studies.

#### 3.1.5. The Effects of Ultrasound Power

Ultrasound powers ranging from 360 to 600 W were chosen to study the extraction efficiency. Meanwhile, other extraction parameters remained constant as follows: ethanol concentration of 70%, L/S ratio of 20 mL/g, extraction time of 30 min, and extraction temperature of 40 °C. As shown in Figure 1e, the ABTS value increased with the ultrasonic power and peaked at 420 W. After that, the antioxidant activity exhibited a downward tendency as the ultrasound power increased. The results indicated that the higher ultrasound power disintegrated the cell walls due to the ultrasonic cavitation, accelerating the release of the antioxidants into the medium. However, the ABTS value decreased as the ultrasound power continued to rise since the excessive ultrasonic power would cause the degradation of the antioxidants [27]. Thus, 420 W was chosen for the following studies.

### 3.2. Response Surface Methodology

#### 3.2.1. Response Surface Design and Experimental Results

Based on the results of the single-factor experiments, the concentration of ethanol, extraction temperature, and ultrasound power were essential parameters affecting the extraction yield of antioxidants from kiwifruit. Therefore, these three parameters were chosen as independent variables, in which *X*_1_ was the concentration of ethanol, *X*_2_ was the extraction temperature, and *X*_3_ was the ultrasound power for the RSM optimization. Meanwhile, other extraction parameters were controlled at the optimal level (L/S ratio of 20 mL/g and extraction time of 30 min). A three-factor, three-level Box–Behnken design was applied to the RSM optimization. Table 1 displays the coded levels of 3 independent variables, and Table 2 summarizes the 17 experimental designs and their results, with the response (ABTS value) ranging from 56.37 to 71.38 μmol Trolox/g DW.

#### 3.2.2. Fitting the Model

The results of the ANOVA, which was carried out to evaluate the quality of the response surface quadratic model, are shown in Table 3. The F-value was high (F = 18.89), and the *p*-value was low (*p* < 0.001), which indicated that the model was statistically significant. Furthermore, both the determination coefficient (R^2^) and the adjusted determination coefficient (adjusted R^2^) were higher than 0.9, which implied that the actual and predicted ABTS values were in a high degree of correlation. The lack of fit value was higher than 0.05, indicating that the established model was valid. The linear parameter (X_1_), interactive parameter (X_2×3_), and the quadratic parameter (X_1_^2^, X_2_^2^, and X_3_^2^) showed significant effects on the yield of antioxidants, while other coefficients (X_2_, X_3_, X_1_X_2_, and X_1_X_3_) were considered insignificant.

Multiple regression analysis was carried out on the experimental results. A second-order polynomial model, which was obtained to analyze the response and three independent variables (coded values), was as follows:

(2) $$Y=70.22-2.16X_{1}+1.08X_{2}-0.64X_{3}+0.37X_{1}X_{2}-0.46X_{1}X_{3}+2.67X_{2}X_{3}-4.60X_{1}{}^{2}-2.22X_{2}{}^{2}-6.65X_{3}{}^{2}$$

In Equation (2), Y indicates the ABTS value of the extracts, and X_1_, X_2_, and X_3_ mean ethanol concentration, extraction temperature, and ultrasound power, respectively.

#### 3.2.3. Model Analysis

**Figure 2:**
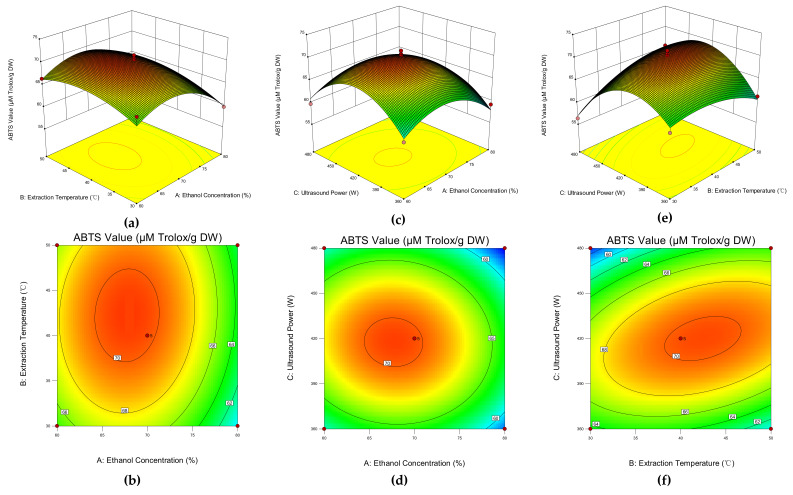
Response surface analysis of different interactions between ethanol concentration and extraction temperature (**a**,**b**); ethanol concentration and ultrasound power (**c**,**d**); and extraction temperature and ultrasound power (**e**,**f**).

Figure 2 exhibits the three-dimensional surface and the contour plot of the model, which visualized the interactive effect of three selected variables on the ABTS value of the extracts. Figure 2a,b shows the effect of the interaction between ethanol concentration (X_1_) and extraction temperature (X_2_) with a fixed ultrasound power of 420 W. As the ethanol concentration increased, the antioxidant capacity increased initially and then decreased as the concentration continued to go up, whereas there was limited impact on the ABTS value caused by the increase of the extraction temperature. Figure 2c,d exhibits the interactive effect between ethanol concentration (X_1_) and ultrasound power (X_3_) at a controlled extraction temperature (40 °C). Both the ethanol concentration and the ultrasonic power had a similar effect on antioxidant activity, which increased the parameters initially and decreased after reaching the peak. However, the effect of ultrasound power on antioxidant capacity was slightly greater than that of ethanol concentration. In Figure 2e,f, the interaction effect between the extraction temperature (X_2_) and the ultrasound power (X_3_) was presented when the ethanol concentration was fixed at 70%. The ABTS value rose stably as the ultrasound power increased and dropped dramatically after reaching the peak, while the ethanol concentration only had a mild influence on the antioxidant capacity. According to the combination of ANOVA results and the response surface plot, the interaction effect between extraction temperature and ultrasound power (X_2_X_3_) was statistically significant. However, the interactive effects between ethanol concentration and extraction temperature (X_1_X_2_) and ethanol concentration and ultrasound power (X_1_X_3_) were non-significant. Furthermore, the combination also revealed that ultrasound power was the most significant parameter that affected the ABTS value, followed by ethanol concentration and extraction temperature.

#### 3.2.4. Verification of the Optimal Extraction Condition

According to the regression equation (Equation (2)), the optimal conditions were as follows: ethanol concentration of 68%, L/S ratio of 20 mL/g, extraction time of 30 min, extraction temperature of 42 °C, and ultrasound power of 420 W. Under these extraction conditions, the predicted ABTS value was 70.58 μM Trolox/g DW. The actual value of ABTS, which was obtained by conducting a single experiment under the optimal conditions, was 70.38 ± 1.38 μM Trolox/g DW, in agreement with the predicted range. The good fitness between the actual and predicted values demonstrated that the RSM model was reliable for predicting optimal antioxidant capacity. In comparison with previous research, the antioxidant capacity of ‘Jinfeng’ kiwifruit was nearly twice as high as that of SunGold (39.31 ± 5.50 µM Trolox/g DW) and Sweet Green (37.18 ± 2.73 µM Trolox/g DW) reported previously [26].

### 3.3. Comparison of Extraction Methods

CSE was carried out to prove that the addition of ultrasound treatment enhanced the extraction efficiency of kiwifruit. The extraction conditions and the ABTS values of two extraction methods are presented in Table 4. The ABTS value of UAE was 18.5% higher than that of CSE (59.39 ± 1.40 μM Trolox/g DW), which could be attributed to ultrasound cavitation. The ultrasonic wave could disintegrate the cell walls and enhance the mass transfer rate, contributing to the diffusion of the antioxidants into the matrix under the same extraction time. In a comparative study by [18], the extracts of common bean obtained by UAE exhibited an eight-fold FRAP value and a seven-fold DPPH value over the extracts obtained by CSE. Another study also reported that antioxidants from *Gordonia axillaris* fruits extracted by UAE for 59.47 min were 2.26 times and 3.62 times greater than those extracted by maceration for 2 h and Soxhlet extraction for 4 h, respectively, indicating that UAE reduced the extraction time as well as enhancing the extraction yield [39]. In addition, UAE could also be used in combination with other techniques, such as the use of deep eutectic solvents and enzymes to improve extraction efficiency [40,41].

### 3.4. Total Phenolic and Flavonoid Contents of Kiwifruit

The TPC and TFC of this kiwifruit cultivar under the optimal extraction conditions were 15.50 ± 0.08 mg GAE/g DW and 5.10 ± 0.09 mg CE/g DW, respectively. According to previous reports, the TPC and TFC of *A. chinensis* were about 4.70–16.52 mg GAE/g DW and 0.27 mg CE/g DW, respectively [1,26]. ‘Jinfeng’ kiwifruit exhibited relatively higher TPC and significantly higher TFC as compared to many other *A. chinensis*, which should be, at least in part, contributed to its antioxidant capacity.

### 3.5. Identification of Antioxidant Phytochemicals in Kiwifruit

UPLC–MS/MS analysis was performed to identify the antioxidant phytochemicals in kiwifruit. In this study, 20 compounds were tentatively identified from ‘Jinfeng’ kiwifruit, including 2 vitamins, 5 non-phenolic organic acids, 5 phenolic acids and their derivatives, 4 flavan-3-ols, 2 flavonols, and 2 coumarins. The results of the tentative identification are shown in Table 5. Among the 20 tentatively identified compounds, 19 compounds, including ascorbic acid, D-pantothenic acid, fumaric acid, malic acid, citric acid, succinic acid, protocatechuic acid-*O*-hexoside, neochlorogenic acid, chlorogenic acid, caffeic acid, caffeic acid-*O*-hexoside, esculin, esculetin, catechin, epicatechin, procyanidin dimer B-type isomers, quercetin 3-*O*-glucoside, and quercetin 3-*O*-rhamnoside, have been reported in other kiwifruit samples [16,42,43,44,45,46]. Itaconic acid was tentatively identified for the first time.

Vitamins are a group of organic compounds with biological activities, and they are beneficial and necessary for human health [47]. In this study, two vitamins were tentatively identified. Compound **1** exhibited the precursor ion at m/z 175 and obtained a fragment ion at m/z 85 by losing the –C_2_H_4_O_2_. According to the previous literature, compound **1** was tentatively identified as ascorbic acid, also known as vitamin C [47]. For compound **7**, daughter ion m/z 88 ([C_3_H_6_NO_2_]^−^) was generated from the precursor ion at m/z 218. Thus, it was tentatively identified as D-pantothenic acid, which is also known as vitamin B5, after comparing it with the database and literature [48]. Ascorbic acid and pantothenic acid are important vitamins in kiwifruit, which vary in concentrations in different kiwifruit species and cultivars [16].

Compounds **2**–**6** were identified as non-phenolic organic acids. Compounds **2**, **4**, and **6** produced [M–H]^−^ ions at m/z 115, 129, and 117, respectively. The fragment ions of these three compounds were generated by losing the –CO_2_ group. Therefore, they were tentatively identified as fumaric, itaconic, and succinic acids [49,50,51]. Compound **3** lost an –H_2_O group from the precursor ion m/z 133 and was tentatively identified as malic acid [49]. Compound **5** produced the [M–H]^−^ at m/z 191 and generated a secondary fragment at m/z 111 by losing –2H_2_O–CO_2_. Compared with the database and previous literature, this compound was tentatively identified as citric acid [49]. Fumaric acid, malic acid, citric acid, and succinic acid were also reported in ‘Jinshi 1’ kiwifruit (*Actinidia chinensis*) previously, and their content varied dynamically in diverse development stages [52]. Itaconic acid was tentatively identified for the first time. Itaconic acid was produced via fermentation, which is seldom reported in the fruit [53]. Thus, further analysis was required for this compound.

Phenolic acid is a group of organic acids containing a phenolic ring, which has a C6–C1 skeleton or C6–C3 skeleton [46]. Compounds **8**, **9**, **12**, **14**, and **15** were tentatively identified as protocatechuic acid-*O*-hexoside, neochlorogenic acid, caffeic acid-*O*-hexoside, caffeic acid, and chlorogenic acid, respectively. Compound **14** produced the [M–H]^−^ at m/z 179 and generated a secondary fragment at m/z 135 by losing a carboxyl group. Compared with the existing literature, this compound was identified as caffeic acid [25,54]. Compound **12** had the [M–H]^−^ at m/z 341 on the MS with two fragment ions at m/z 179 ([M–H–hexoside]^−^) and m/z 135 ([M–H–hexoside–COOH]^−^) in the MS^2^ spectrum. Two daughter ions were the same as the fragments produced in caffeic acid; thus, compound **12** was regarded as a caffeic acid derivative and tentatively identified as caffeic acid-*O*-hexoside [55]. Two caffeic acid-*O*-hexosides were reported previously and exhibited relatively higher contents than other determined phenolic compounds [56]. Compounds **9** and **15** shared the same [M–H]^−^ ion at m/z 353 and similar MS^2^ fragment ions at m/z 191, 179, and 135 but varied in retention time. Analysis on the MS^2^ spectrum, the generation of m/z 191 ([C_7_H_11_O_6_]^−^) fragment was caused by losing the caffeoyl, while the generation of m/z 179 ([C_9_H_8_O_4_]^−^) fragment was caused by losing the quinoyl group. The generation of m/z 135 contributed to the separation of –COOH from the m/z 179 fragments. When compared to previous findings, two compounds were identified as neochlorogenic acid and chlorogenic acid [25,55]. Chlorogenic acid and its two isomers, neochlorogenic acid and cryptochlorogenic acid, are important phenolic acids in kiwifruit and have been reported in plenty of studies [1,57]. However, the presence and the contents of these three compounds depends on the species and cultivar of the kiwifruit as well as the development stage [16]. The deprotonated ion of compound **8** was at m/z 315, and two fragments were at m/z 153 and m/z 109. Ion m/z 153 ([C_7_H_6_O_4_]^−^) was generated by losing the hexoside from the precursor ion, and the ion m/z 109 ([C_6_H_5_O_2_]^−^) was generated by losing the –COOH from ion m/z 153. According to previous findings, compound **8** was tentatively identified as protocatechuic acid-*O*-hexoside, which could also be called 3,4-dihydroxybenzoic acid-*O*-hexoside [55]. Previous literature has reported that 3-hydroxy-4-O-β-D-glucopyranoside was identified in the extract of kiwifruit root [46].

**Table 5:** Tentative identification of phytochemical compounds in kiwifruit by using UPLC–MS/MS.

No.	RT (min)	[M–H]^−^	MS^2^ Ion Fragments (m/z)	Formula	Identified Compounds	References
**1**	0.90	175	115, 87	C_6_H_8_O_6_	Ascorbic acid	[47]
**2**	0.98	115	71	C_4_H_4_O_4_	Fumaric acid	[49,51]
**3**	1.01	133	115	C_4_H_6_O_5_	Malic acid	[49,51]
**4**	1.17	129	85	C_5_H_6_O_4_	Itaconic acid	[50]
**5**	1.3	191	111	C_6_H_8_O_7_	Citric acid	[49]
**6**	1.38	117	73	C_4_H_6_O_4_	Succinic acid	[49,51]
**7**	2.61	218	88	C_9_H_17_NO_5_	D-Pantothenic acid	[48]
**8**	3.17	315	153	C_13_H_16_O_9_	Protocatechuic acid-*O*-hexoside	[46,55]
**9**	3.80	353	191, 179, 135	C_16_H_18_O_9_	Neochlorogenic acid	[25,55]
**10**	4.97	339	177	C_15_H_18_O_9_	Esculin	[58]
**11**	7.16	289	245, 179, 205	C_15_H_14_O_6_	Catechin	[25,46]
**12**	7.26	341	179, 135	C_15_H_18_O_9_	Caffeic acid-*O*-hexoside	[55]
**13**	7.90	177	133	C_9_H_6_O_4_	Esculetin	[46]
**14**	8.75	179	135	C_9_H_8_O_4_	Caffeic acid	[25,54]
**15**	10.07	353	191, 179, 135	C_16_H_18_O_9_	Chlorogenic acid	[25,55]
**16**	11.07	577	407, 289, 245	C_30_H_26_O_12_	Procyanidin dimer B-type isomer	[46,59]
**17**	11.65	577	407, 289, 245	C_30_H_26_O_12_	Procyanidin dimer B-type isomer	[46,59]
**18**	13.69	289	245, 179, 125	C_15_H_14_O_6_	Epicatechin	[25,46,55]
**19**	26.44	463	301, 151	C_21_H_20_O_12_	Quercetin 3-*O*-glucoside	[46,55]
**20**	29.33	447	301, 151	C_21_H_20_O_11_	Quercetin 3-*O*-rhamnoside	[46,55]

Coumarin is a group of phenolic compounds exhibiting strong antioxidant and anti-inflammatory capacities [58,60]. Two coumarins, esculin and esculetin, were identified from this kiwifruit cultivar. Compound **13** was recognized as esculetin as it lost the –CO_2_ from the precursor ion (m/z 177) [46]. Compound **10** was identified as esculin, which is also known as 6,7-dihydroxycoumarin-6-glucoside. The daughter ion of this compound was generated by losing the aglycone from the parental ion [46,58].

Flavan-3-ol is a large group of flavonoids with a symbolic ion fragment at m/z 289, comprising catechin and epicatechin and their polymers and glycosides [46]. Four flavan-3-ols were tentatively identified, including catechin, epicatechin, and two procyanidin dimer B-type isomers. Compounds **11** and **18** produced the same [M–H]^−^ ion at m/z 289 and similar MS^2^ fragment ions at m/z 245, m/z 179, and 271 but varied in retention time. The ion m/z 245 ([C_14_H_13_O_4_]^−^) fragment was generated through losing the CO_2_, while the generation of the m/z 179 ([C_9_H_8_O_4_]^−^) fragment was caused by losing hydroquinone (–C_6_H_6_O_2_). The fragment at m/z 271 was obtained by dehydrating from the [M–H]^−^ ion. According to previous findings, compound **11** was catechin, and compound **18** was epicatechin since the retention time of compound **11** was shorter than that of compound **18** [25,46,55]. Compounds **16** and **17** had the same deprotonated molecular ion at m/z 577. Three major daughter ions were obtained at m/z 407, m/z 289, and m/z 245, respectively. The generation of MS^2^ ion at m/z 407 could be due to the dehydration from the [C_22_H_17_O_9_]^−^ ion. Quinone–methide cleavage from the deprotonated molecular ion produced fragment ion at m/z 289 and then derived the ion m/z 245 by losing a carboxyl group. Thus, compounds **16** and **17** were identified as procyanidin dimer B-type isomers [46,59].

Flavonol is another significant group of flavonoids, consisting of kaempferol, quercetin, isorhamnetin, and their glycosides [46]. Ion m/z 301 is a typical fragment ion of quercetin derivatives [46]. Since compounds 19 and 20 had this typical ion fragment, they were considered quercetin derivatives. The generation of ion m/z 301 represented that they lost their respective glycosides (glucoside and rhamnoside), and another ion fragment with m/z 151 was subsequently generated by losing a –C_8_H_6_O_3_. Compared with the database and previous literature, compound **19** was identified as quercetin 3-*O*-glucoside, and compound **20** was determined as quercetin 3-*O*-rhamnoside [46,55]. However, kaempferol, its derivatives, and quercetin, which were identified in ‘Hayward’ kiwifruit, were not identified in the current study [57].

### 3.6. Quantification of Ascorbic Acid and Phenolic Compounds in Kiwifruit

**Figure 3:**
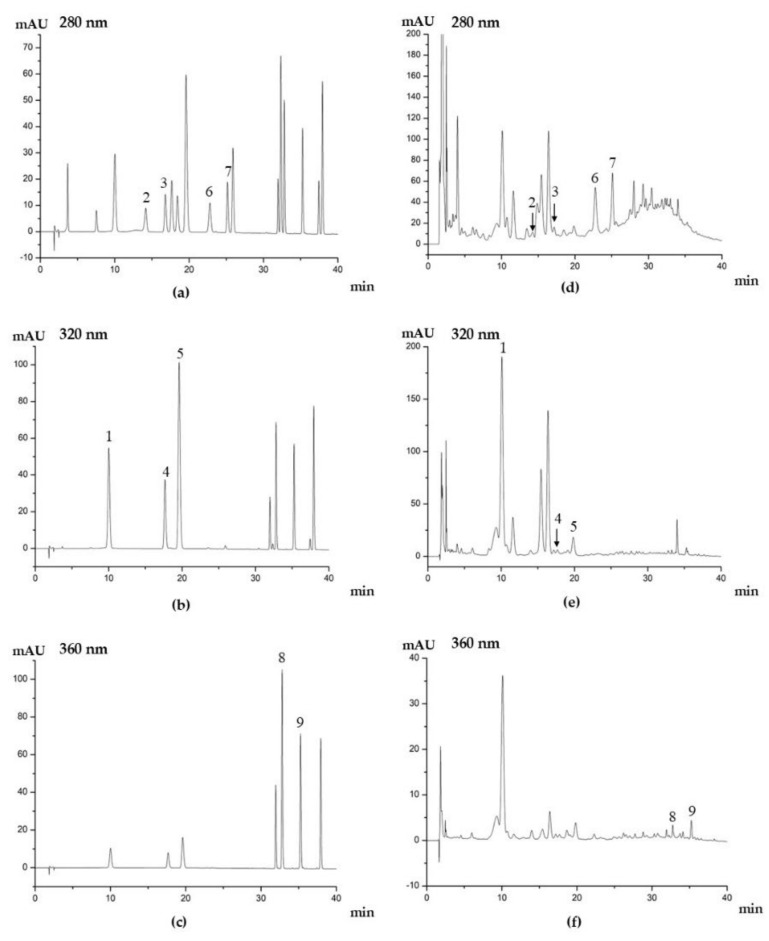
HPLC chromatograms mixed standards (**a**–**c**) and phenolic profiles ‘Jinfeng’ kiwifruit (**d**–**f**). 1, neochlorogenic acid; 2, procyanidin B1; 3, catechin; 4, chlorogenic acid; 5, caffeic acid; 6, procyanidin B2; 7, epicatechin; 8, quercetin-3-*O*-glucoside; 9, quercetin-3-*O*-rhamnoside. Detection was made at 280 nm for hydroxybenzoic acids and flavanols, 320 nm for hydroxycinnamic acids, and 360 nm for flavonols.

The determination of ascorbic acid and major phenolic compounds in kiwifruit was conducted using HPLC–DAD by comparing the retention time and the calibration curves with those of standard compounds. The results are displayed in Table 6. Figure 3 shows the chromatograms of the mixed standards and the phenolic profiles of ‘Jinfeng’ kiwifruit. The content of ascorbic acid of ‘Jinfeng’ kiwifruit was 7.21 ± 0.08 mg/g DW, which was 20% higher than that of ‘Hayward’ kiwifruit (*Actinidia deliciosa*) (5.63 mg/g DW), and 40% higher than that of other *Actinidia chinensis* cultivars (4.27–4.34 mg/g dry mass) [61,62]. The differences in ascorbic acid content in kiwifruit might be due to the diversity of species and cultivars [16]. For example, the ascorbic acid content of 14 kiwifruit cultivars from 5 species varied widely, ranging from 51.32 to 390.68 mg/100 g FW, with the most abundant ascorbic acid found in ‘Hongshi’ kiwifruit from *Actinidia chinensis*, while *Actinidia arguta* had the lowest level of ascorbic acid [1].

In total, nine major phenolic compounds were detected and quantified, including three phenolic acids (neochlorogenic acid, chlorogenic acid, and caffeic acid), four flavanols (procyanidin B1, procyanidin B2, catechin, and epicatechin), and two flavonols (quercetin 3-*O*-glucoside and quercetin 3-*O*-rhamnoside). Among these nine compounds, procyanidin B2, neochlorogenic acid, and epicatechin were the three most abundant phenolic compounds with contents over 100 μg/g DW. Similar phenolic profiles were also reported in other yellow-fleshed kiwifruit cultivars [1,63]. Neochlorogenic acid was the most abundant phenolic acid in ‘Jinfeng’ kiwifruit, with the content at 119.90 ± 1.73 µg/g DW, which was slightly lower than that of ‘Jinlong’ kiwifruit (133.72 ± 3.98 µg/g DW) but higher than that of ‘Jinshi’, ‘Jinyan’, and ‘Hort 16A’ (107.06 ± 1.74, 6.29 ± 0.11, and 17.78 ± 1.53 µg/g DW, respectively) reported previously [1]. Unlike its isomer, the content of chlorogenic acid in ‘Jinfeng’ kiwifruit was much lower than that of neochlorogenic acid. The ratios of neochlorogenic acid content to chlorogenic acid content could be affected by cultivar, such that ‘Jinshi’ and ‘Jinlong’ had higher neochlorogenic acid contents, while ‘Jinyan’ and ‘Hort 16A’ exhibited higher chlorogenic acid levels [1]. Caffeic acid was a common phenolic acid found in kiwifruit, even though its content was less than neochlorogenic acid. The content of caffeic acid of ‘Jinfeng’ kiwifruit was 6.93 ± 0.40 µg/g DW, which was higher than of ‘Hort 16A’ (0.04 µg/g DW) [63]. Four flavanols were determined in this research, including procyanidin B1, procyanidin B2, catechin, and epicatechin. The content of procyanidin B2 in ‘Jinfeng’ kiwifruit was 166.67 ± 2.84 µg/g DW, which was much higher than that of its isomer, procyanidin B1. This result was different from a previous study in which the procyanidin B1 contents in four yellow-fleshed cultivars of kiwifruit ranged from 145.68 to 203.68 µg/g DW, which was much higher than that of procyanidin B2. ‘Jinyan’ kiwifruit had the highest content of procyanidin B2 among these four kiwifruit cultivars, with content of 125.38 ± 2.00 µg/g DW, which did not even appear in ‘Jinshi’ kiwifruit [1]. As for epicatechin, the content in ‘Jinfeng’ (110.28 ± 0.50 µg/g DW) was much higher than that in other four yellow-fleshed kiwifruit cultivars (38.98–60.70 µg/g DW) [1] and in ‘Hort 16A’ (5.15 µg/g DW) [63]. Though catechin is an isomer of epicatechin, its content was much less than epicatechin, which was in accordance with previous research [1,56,63]. In this research, only two flavonols were quantified, which were quercetin 3-*O*-glucoside and quercetin 3-*O*-rhamnoside. ‘Jinfeng’ kiwifruit had a lower level of quercetin 3-*O*-rhamnoside than ‘Jinshi’ (4.59 µg/g DW) and ‘Jinyan’ (4.73 µg/g DW), while it had a higher level of quercetin 3-*O*-glucoside than ‘Hort 16A’ (0.45 µg/g DW) [1,63]. The content of most phenolic compounds in ‘Jinfeng’ kiwifruit was higher than that in other yellow-fleshed kiwifruit reported before [1,63], which means ‘Jinfeng’ kiwifruit might have a higher antioxidant capacity than other yellow-fleshed kiwifruit cultivars.

## 4. Conclusions

Ultrasound-assisted extraction has been optimized for recovering phytochemicals from ‘Jinfeng’ kiwifruit, and it resulted in an 18.5% increment in antioxidant activity compared with conventional extraction. Twenty phytochemicals were tentatively identified by UPLC–MS/MS, and one of them was identified for the first time and needed further confirmative study. In addition, nine phenolic compounds were quantified by HPLC–DAD, and procyanidin B2, neochlorogenic acid, and epicatechin were the three most abundant phenolic compounds. Though ‘Jinfeng’ kiwifruit showed antioxidant profiles similar to those of other kiwifruit cultivars, it exhibited a relatively high level of TPC, TFC, and ascorbic acid content. This work reveals the high antioxidant capacity of ‘Jinfeng’ kiwifruit and its major antioxidants. However, evaluations of the nutritional composition and other health benefits of this kiwifruit cultivar are limited, which require further study. In addition, the effects of different developmental stages and processing techniques on its nutritional value should be studied. Collectively, ‘Jinfeng’ kiwifruit is rich in antioxidant phytochemicals, can be a good source of dietary antioxidants, and can also be developed into functional foods with potential health benefits, which should be further investigated in the future.

## Figures and Tables

**Table 1:** Coded levels of the independent variables.

Independent Variables	Coded Units	Coded Levels		
		−1	0	1
Ethanol concentration (%)	X_1_	60	70	80
Extraction temperature (°C)	X_2_	30	40	50
Ultrasound Power (W)	X_3_	360	420	480

**Table 2:** Response surface design and ABTS values of the extracts.

Run	X_1_ (%)	X_2_ (°C)	X_3_ (W)	Y (μM Trolox/g DW)	
				Experimental Data	Predicted Results
1	0(70)	1(50)	1(480)	65.54 ± 0.50	64.46
2	0(70)	0(40)	0(420)	69.26 ± 0.25	70.22
3	1(80)	1(50)	0(420)	60.88 ± 0.81	62.69
4	0(70)	0(40)	0(420)	69.56 ± 0.67	70.22
5	1(80)	0(40)	−1(360)	59.13 ± 1.31	57.91
6	−1(60)	0(40)	−1(360)	60.57 ± 0.82	61.30
7	−1(60)	0(40)	1(480)	59.72 ± 1.44	60.95
8	−1(60)	1(50)	0(420)	66.41 ± 0.39	66.27
9	1(80)	−1(30)	0(420)	59.64 ± 1.16	59.79
10	1(80)	0(40)	1(480)	56.44 ± 1.05	55.70
11	0(70)	0(40)	0(420)	70.24 ± 1.60	70.22
12	0(70)	0(40)	0(420)	70.66 ± 1.27	70.22
13	0(70)	−1(30)	−1(360)	62.50 ± 1.77	63.58
14	0(70)	1(50)	−1(360)	60.99 ± 2.00	60.40
15	0(70)	0(40)	0(420)	71.38 ± 1.26	70.22
16	−1(60)	−1(30)	0(420)	66.67 ± 0.39	64.85
17	0(70)	−1(30)	1(480)	56.37 ± 2.48	56.96

DW, dry weight; μmol Trolox/g DW, micromoles Trolox equivalents per gram dry weight. Data are expressed as mean values ± standard deviation (*n* = 3).

**Table 3:** ANOVA for the response surface quadratic model.

Effects	Source	Sum of Square	df	Mean Square	F-Value	*p*-Value Prob > F
Total effect	Model	404.03	9	44.89	18.89	0.0004 ^a^
Linear effect	X_1_	37.36	1	37.36	15.72	0.0054 ^a^
	X_2_	9.33	1	9.33	3.92	0.0880
	X_3_	3.27	1	3.27	1.38	0.2790
Interactive effect	X_1_X_2_	0.56	1	0.56	0.23	0.6435
	X_1_X_3_	0.86	1	0.86	0.36	0.5671
	X_2_X_3_	28.53	1	28.53	12.01	0.0105 ^a^
Quadratic effect	X_1_^2^	89.22	1	89.22	37.54	0.0005 ^a^
	X_2_^2^	20.70	1	20.70	8.71	0.0214 ^a^
	X_3_^2^	186.30	1	186.30	78.39	<0.0001 ^a^
	Residual	16.64	7	2.38		
	Lack of Fit	13.74	3	4.58	6.34	0.0533
	Pure Error	2.89	4	0.72		
	Corrected Total	420.66	16			
	R^2^	0.9605				
	Adjusted R^2^	0.9096				

^a^ Stands for statical significance (*p* < 0.05).

**Table 4:** ABTS value comparison between ultrasound-assisted extraction (UAE) and conventional solvent extraction (CSE).

Extraction Method	Ethanol Concentration	L/S Ratio	Extraction Time	Extraction Temperature	Ultrasonic Power	ABTS Value (μM Trolox/g DW)
UAE	68%	20:1	30 min	42 °C	420 W	70.38 ± 1.38
CSE	68%	20:1	30 min	42 °C	None	59.39 ± 1.40

DW, dry weight; μM Trolox/g DW, μM Trolox equivalents per g dry weight.

**Table 6:** The contents of vitamin C and major phenolic compounds.

Compounds	Regression Equation	Linear Range (µg/mL)	Correlation Coefficient (R^2^)	Retention Time (min)	Content
Vitamin (mg/g DW)					
Ascorbic acid	Y = 26.536X + 81.753	10.00–100.00	0.9971	2.0	7.21 ± 0.08
Phenolic compounds (µg/g DW)					
Neochlorogenic acid	Y = 38.141X − 126.62	5.88–29.41	0.9980	9.9	119.90 ± 1.73
Procyanidin B1	Y = 6.1598X − 7.2701	5.88–29.41	0.9984	14.1	15.93 ± 0.30
Catechin	Y = 8.5506X − 9.5818	5.88–29.41	0.9997	17.1	16.68 ± 0.34
Chlorogenic acid	Y = 39.002X − 66.271	3.53–17.65	0.9984	17.6	3.53 ± 0.17
Caffeic acid	Y = 66.056X − 82.779	5.88–29.41	0.9996	19.8	6.93 ± 0.40
Procyanidin B2	Y = 8.1696X − 11.262	5.88–29.41	0.9991	22.7	166.67 ± 2.84
Epicatechin	Y = 8.7362X − 5.9765	5.88–29.41	0.9998	25.1	110.28 ± 0.50
Quercetin 3-*O*-glucoside	Y = 31.11X − 33.905	5.88–29.41	0.9997	32.7	2.04 ± 0.01
Quercetin 3-*O*-rhamnoside	Y = 21.924X − 26.664	5.88–29.41	0.9992	35.2	3.37 ± 0.06

X, concentration (µg/mL); Y, peak area. Data are expressed as mean values ± standard deviation (*n* = 3).
